# The Effectiveness of Functional Electrical Stimulation Based on a Normal Gait Pattern on Subjects with Early Stroke: A Randomized Controlled Trial

**DOI:** 10.1155/2014/545408

**Published:** 2014-07-10

**Authors:** Zhimei Tan, Huihua Liu, Tiebin Yan, Dongmei Jin, Xiaokuo He, Xiuyuan Zheng, Shuwei Xu, Chunmei Tan

**Affiliations:** ^1^Department of Rehabilitation Medicine, Sun Yat-sen Memorial Hospital, Sun Yat-sen University, 107 Yanjiang West Road, Guangzhou 510120, China; ^2^Department of Rehabilitation Medicine, The First Affiliated Hospital of Zhengzhou University, 1 Jianshe East Road, Zhengzhou, China; ^3^Henan Provincial Technology Center of Rehabilitation and Assistive Devices, 13 Jinger Road, Zhengzhou, China

## Abstract

*Objective.* To investigate the effectiveness of four-channel FES based on a normal gait pattern on improving functional ability in subjects early after ischemic stroke. * Methods.* Forty-five subjects were randomly assigned into a four-channel FES group (*n* = 16), a placebo group (*n* = 15), or a dual-channel group (*n* = 14). Stimulation lasted for 30 min in each session with 1 session/day, 5 days a week for 3 weeks. All subjects were assessed at baseline, at 3 weeks of treatment, and at 3 months after the treatment had finished. The assessments included Fugl-Meyer Assessment (FMA), the Postural Assessment Scale for Stroke Patients (PASS), Berg Balance Scale (BBS), Functional Ambulation Category (FAC), and the Modified Barthel Index (MBI). * Results.* All 3 groups demonstrated significant improvements in all outcome measurements from pre- to posttreatment and further gains at followup. The score of FMA and MBI improved significantly in the four-channel group at the end of the 3 weeks of training. And the scores of PASS, BBS, MBI, and FAC in the four-channel group were significantly higher than those of the placebo group. * Conclusions.* This study indicated that four-channel FES can improve motor function, balance, walking ability, and performance of activities of daily living in subjects with early ischemic stroke.

## 1. Introduction

Stroke is the leading cause of long-term disability. Most survivors demonstrate motor dysfunction and inability to ambulate in the acute phase, and gait impairments can remain 3 months after stroke [1, 2]. About 80% of survivors might ultimately recover the ability to walk for significant distances [2], but they often have serious residual deficits such as slow gait [3, 4], foot drop [5], and gait asymmetry [4] which severely affect their ability to walk. Restoring nearly normal walking has therefore been a major goal of rehabilitation for poststroke subjects.

Functional electrical stimulation (FES) was first applied in stroke rehabilitation by Liberson and his colleagues [6] in 1961. It successfully corrected foot drop when delivered only to the ankle dorsiflexors in the chronic phase. Since then, numerous studies have shown that FES is an effective treatment for improving motor function [7, 8], increasing walking speed [8], and reducing spasticity [8, 9] after stroke. However, most FES devices are single- or dual-channel and are used to stimulate ankle dorsiflexion alone, thereby failing to improve gait deficits at the knee and hip. Although FES stimulates ankle dorsiflexion and can correct foot drop, it also decreases knee flexion and ankle plantarflexion at toe-off in the swing phase of gait [10] which decreases the propulsive force generated at the transition from the stance phase to the swing phase. In fact, several studies have demonstrated that there is high correlation between the strength of the knee extensors and flexors, the propulsive force generated through ankle plantarflexion, and gait performance [11–13]. Multichannel FES has improved this situation. It can be applied to a group of muscles rather than to the ankle dorsiflexors alone. A study led by Stanic [14] has shown that multichannel FES given 10 to 60 minutes per day, 3 times per week for 1 month, improved the gait performance of hemiplegic subjects. Bogataj and his colleagues [15] have applied multichannel FES to activate the lower limb muscles of 20 chronic hemiplegics. After daily treatment, 5 days per week for 1 to 3 weeks, subjects who previously had been unable to walk walked again.

Although the positive effects of multichannel and conventional FES on the affected lower extremity have been demonstrated in several studies [14–16], few have addressed time-dependent stimulation based on the normal gait pattern and compared the effects. Also the stimulation in published studies has varied either in each session or in terms of the duration of the studies [14–16].

This study was therefore designed to observe the effects of multichannel FES based on a normal gait pattern, meanwhile comparing those with the conventional stimulating ankle dorsiflexion alone.

## 2. Patients and Methods

### 2.1. Study Design

This was a single-blind, stratified, randomized, controlled trial. In the study, the single-blind way referred to that all subjects were blinded to enter the research and receive FES therapy, but the therapists were not. Randomization was using Jensen's [17] computerized minimization method, which generated a random number each time. The number represented each subject's specific group. The subjects were randomly assigned to either the four-channel group, the four-channel placebo group, or the dual-channel group. In order to minimize the possibility of inappropriate grouping of covariant, the sample was stratified by age (45 to 60 years and 60 to 80 years), gender (male and female), and Brunnstrom stage (I, II, or IV). Research of small sample size was firstly conducted in accordance with the designed method to fix the feasibility of study and estimated the sample size, and the sample size was calculated by using Power Analysis of Sample Size Software (PASS 2008). The optimum sample size was determined to be 52 in total with a minimum of 15 in each of the 3 groups on the basis of the score of FMA with an *α* level of 0.05 and power of 0.9. The study protocol was approved by the ethics committee of Sun Yat-sen Memorial Hospital. All participants gave informed written consent to their participation.

### 2.2. Participants

**Figure 1:**
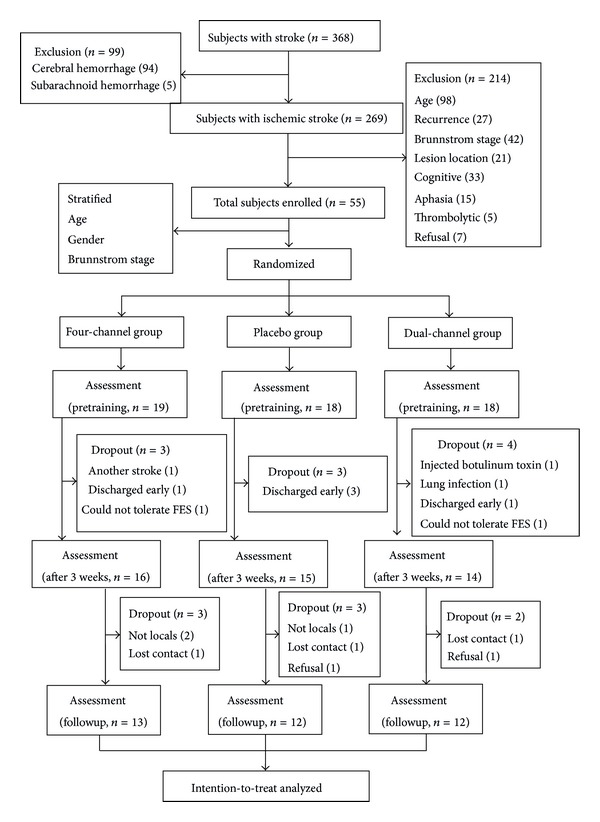
Flowchart of the study.

From May 2011 to April 2012, 368 subjects with stroke were recruited from the rehabilitation medicine departments of Sun Yat-sen Memorial Hospital, Sun Yat-sen University, and the Guangdong Second Provincial Traditional Chinese Medicine Hospital (Baiyun branch). Inclusion criteria were a first ischemic stroke diagnosed by either computed tomography or magnetic resonance imaging, unilateral hemiparalysis, ages 45 to 80 years old, within 3 months postonset, and a Brunnstrom stage of I, II, or IV. Exclusion criteria were progressive ischemic stroke, cerebellum or brain stem lesions, thrombolysis or embolectomy, severe aphasia or hypoesthesia, cognitive dysfunction (indicated by scoring less than 7 on the Abbreviated Mental Test [18]), heart, lung, liver, or kidney comorbidity, traumatic brain injury, cancer, and refusal to give informed consent.  Figure 1 shows the flow of subject recruitment.

**Figure 2:**
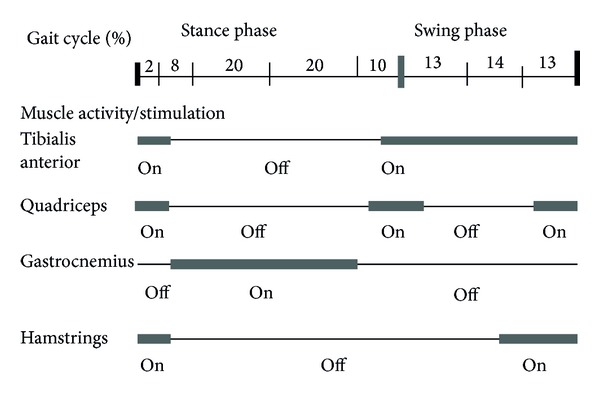
Timing of stimulation sequence in a gait cycle.

The four-channel FES system used in the study was designed by Sun Yat-sen Memorial Hospital, Sun Yat-sen University. The system consists of a stimulating unit and four sets of two stimulation electrodes. The system has two stimulation programmes. Programme 1 delivers stimulation through the four channels. The two electrodes of each channel are placed to stimulate the tibialis anterior, quadriceps, hamstrings, and gastrocnemius of the affected leg [19], respectively, and the time of stimulation sequence in a gait cycle was shown in Figure 2. Programme 2 combines two of the four channels to stimulate the tibialis anterior and the peroneus longus and brevis to activate ankle dorsiflexion and eversion, and the stimulation sequence was synchronous. Square electrodes (4 cm × 4 cm) are attached over the motor points of the tibialis anterior, peroneus longus, and peroneus brevis muscles, but rectangular electrodes (9 cm × 6 cm) are attached over the motor points and longitudinal to the quadriceps, biceps femoris, and gastrocnemius. In this study the locations of the motor points were confirmed using peripheral nerve electrical stimulation (PNS) (SY-708A, Tianrui, China).

### 2.3. Intervention

All subjects received the same conventional stroke rehabilitation program including 30 minutes of physical therapy daily based on the neurodevelopmental facilitation approach and 30 minutes of daily occupational therapy focused on ADL training, given 1 session each day, 5 days a week for 3 weeks. Furthermore, many subjects enrolled in the study had the comorbidities following stroke such as hypertension, diabetes, high cholesterol, and transient ischemic attack. The above comorbidities should be effectively controlled to prevent stroke recurrence and affect rehabilitation training.

**Figure 3:**
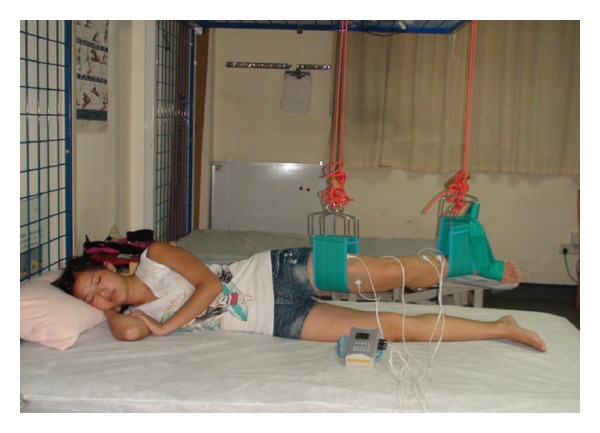
Treatment setting for four-channel functional electrical stimulation in side lying.

Subjects in the four-channel group received four-channel FES therapy stimulating the affected lower extremity to mimic a normal gait cycle with programme 1 (Figure 3). Subjects in the placebo group were also given the four-channel FES therapy, but in fact only the indicator light was working and no current was output; the purpose of this was to allow them believe that they receive the same treatment with other subjects. Those in the dual-channel group received dual-channel FES therapy to activate the affected plantar flexors with programme 2. In both programmes FES was delivered at a frequency of 30 Hz with a pulse width of 200 *μ*s. The current amplitude was adjusted to each patient's maximum tolerable intensity. The gait cycle of the four-channel FES was set at 5 s and that of the dual-channel FES was set at 5 s on and 5 s off. Because most of the subjects could not walk independently, in order to standardize the treatment, all of the subjects were treated in the side-lying position with the affected lower extremity supported by two slings fixed over the knee and ankle joints and the ankle fixed in a neutral position to prevent foot drop. Stimulation lasted for 30 minutes per session, 1 session per day, and 5 days per week for 3 weeks.

### 2.4. Outcome Measure

The following outcomes were assessed at baseline, then weekly during the 3 weeks of treatment in the hospital, and at 3 months followup after the treatment. All subjects were of course blinded to the group to which they had been assigned by the computer, but the physician and therapist were not blinded because of the different positions of the electrodes and the different movement patterns induced by FES or placebo stimulation. To minimize any possible bias during assessment, all subjects were assessed by the same therapist, who had received special training prior to the study. The reliability of the outcome assessments was tested before beginning the study.

The lower extremity of Fugl-Meyer Movement Assessment (FMA) [20] was administered to assess lower extremity motor function. The maximum possible score is 34. The higher the score, the better the motor function of the lower extremity.

The Postural Assessment Scale for Stroke Patients (PASS) [21, 22] and Berg Balance Scale (BBS) [23] were applied to assess functional balance. The PASS mainly assesses posture control in rising from lying to sitting for stroke survivors in the first months. The BBS has excellent predictive validity to assess balance after stroke, but it also has ceiling and floor effects when used to assess subjects who have severe or mild balance impairment [24]. In order to better detect meaningful changes, the PASS and the BBS were combined to detect changes in balance.

Functional Ambulation Category (FAC) [25, 26] was used to assess the subjects' gait performance. A score of 0 represents no walking ability; scores >3 represent walking independently.

The Modified Barthel Index (MBI) [27, 28] was used to assess dependence in the activities of daily living. A score ≥ 60 represents independent living, with lower scores representing assisted or totally dependent living.

### 2.5. Statistical Analysis

Version 16.0 of the SPSS software package was used to analyze the data. Descriptive statistics were presented as mean ± standard deviation. The date of time since stroke, age, and the FMA, PASS, BBS, and MBI results were consistent with the normal distribution and compared by the parametric analysis of variance (ANOVA), whereas gender, Brunnstrom stage, and FAC were compared using *χ* ^2^ tests.

To better reflect the clinical and long-term effectiveness of the treatments, an intention-to-treat design was used to deal with all outcome measurements for subjects who received training for more than 2 weeks but did not finish the study, or who recovered but did not come back for the follow-up assessment. Repeated measures analysis of variance was used to test the significance of the treatment effects observed before, during and after treatment, and at followup within the groups. One-way ANOVA followed by post hoc tests with an LSD correction was used for between-group comparisons. The significance level was set at *P* < 0.05.

## 3. Results

### 3.1. Baseline Characteristics

Of the 368 stroke subjects admitted to the hospitals during the study period, 99 were cerebral hemorrhage victims. From the 269 subjects with ischemic stroke, 214 subjects were excluded because they met one of the exclusion criteria. Fifty-five subjects met the inclusion criteria and were randomly assigned into a group, yielding 19 subjects in the four-channel group and 18 in each of the others. Ten subjects failed to complete the study and received treatment for less than 2 weeks as detailed in Figure 1. Forty-five subjects finally completed the study.  Table 1 shows the characteristics of all the subjects. There were no significant differences among the 3 groups at baseline in terms of age, gender, and time since stroke or the distribution of Brunnstrom stages.

### 3.2. Treatment Results

**Figure 4:**
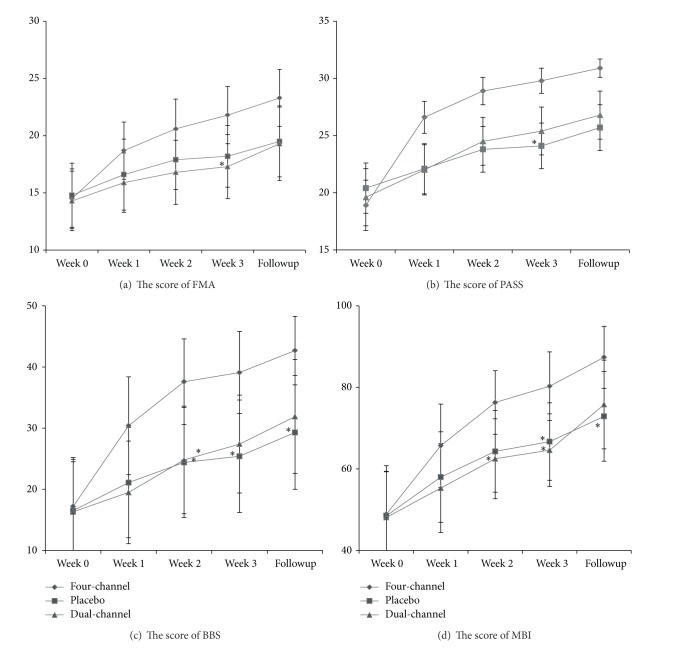
Comparison among the 3 groups: (a) FMA score, (b) PASS score, (c) BBS score, and (d) MBI at weeks 0, 1, 2, 3, and at follow-up 3 months later. ^∗^indicates a difference compared with the four-channel group significant at the 5% level of confidence.

The average FMA lower extremity scores (Table 2) showed no significant differences among the 3 groups at baseline. After 3 weeks of treatment, there was a significant difference in FMA scores between the four-channel and dual-channel groups (*P* = 0.024), but no significant difference between the four-channel and placebo groups (*P* = 0.062) (Figure 4).

There were no significant differences in PASS and BBS scores among the 3 groups at baseline, and after 3 weeks of treatment, the differences still were not significant. But further comparison demonstrated that the difference between the four-channel and placebo groups in PASS (*P* = 0.031) and BBS (*P* = 0.022) scores reached significance. The differences in PASS and BBS scores between the four-channel and dual-channel groups were not, however, significant (Table 2, Figure 4).

The mean initial MBI values were 48.9 ± 23.8, 48.5 ± 21.7, and 48.1 ± 22.6 for the four-channel, placebo, and dual-channel groups, respectively. Those mean scores increased to 80.3 ± 16.5, 66.7 ± 19.1, and 64.6 ± 17.8 after 3 weeks of treatment, 64.2% improvement for the four-channel group, 37.5% for the placebo group, and 34.3% for the dual-channel group. One-way ANOVA analysis showed that the four-channel group had significantly greater improvement compared with the placebo (*P* = 0.039) and dual-channel groups (*P* = 0.021) (Table 2, Figure 4).

At the beginning of the study, 26 of the 45 subjects who eventually completed the study could not walk independently (9/16 in the four-channel group, 9/15 in the placebo group, and 8/14 in the dual-channel group). After 3 weeks of treatment, significant improvement was observed in all 3 groups. Fifty-six percent of the four-channel group was walking independently, compared with 20% in the placebo group and 21% in the dual-channel.

After 3 weeks of treatment, the correlation analysis on possible relationship between changes on motor performance was made, and the results showed that the score of FMA, PASS, and BBS had higher correlation with the score of MBI (*r* = 0.833, 0.836, 0.846, resp., *P* < 0.001).

To compare the long-term effects of FES, all of the measurements were reassessed 3 months after the 3-week treatment had ended. A total of 37 came back, with 13 from the four-channel group and 12 each from the placebo and the dual-channel groups. Eight subjects did not come back (3 had moved out of the city, 3 had lost contact, and 2 refused). Almost all of the subjects in all 3 groups demonstrated further improvement since the 3-week treatment had finished, but significant differences were found only between the four-channel and placebo groups in terms of BBS (*P* = 0.028) and MBI (*P* = 0.047). At that point 68.8% of those in the four-channel group walked independently compared with 40% of those in the placebo group and 35.7% of the dual-channel group.

## 4. Discussion

The novel gait stimulator tested in this study can stimulate four groups of main muscles of the affected lower extremity in a sequence simulating the gait cycle, but to date, only very few studies have reported time-dependent stimulation of groups of lower extremity muscles simulating walking [29, 30]. Most FES systems have been limited to single-channel stimulation, usually of the dorsiflexors. This overlooks the important role of other lower extremity muscles during ambulation as primary controllers of the hip, knee, and ankle complex during the stance and swing phases. In recent years some studies have begun to apply dual-channel FES to activate both the dorsiflexors and plantarflexors [29, 30]. Some have even combined multichannel FES with other gait training technologies (treadmill training, a gait robot, and motorized cycle training) to make the training more physiological [31–33]. The results suggest that compared with conventional FES stimulating ankle dorsiflexion alone, the four-channel FES applied in the study may provide a better alternative rehabilitation approach to improve walking function.

The results show that all of the subjects had significant improvements in all of the outcome measures after 3 weeks of treatment and that the improvements persisted for at least 3 months after the treatment ended. The significant gains suggest that the study protocol was appropriate, and typical stroke patients should be able to tolerate 3 weeks of such training. Though no explicit evidence of subjects' tolerance was collected, none of the severe adverse reactions reported by some investigators [32, 33] were observed.

The between-group comparisons show that the four-channel group achieved significantly better improvements in the FMA and MBI measures than in the dual-channel group at week 3 and better improvements in PASS, BBS, MBI, and FAC than in the placebo group at week 3 and in the 3-month followup. Programmed four-channel FES may thus be a more effective approach which can better improve subjects' functional ability. The lack of any significant difference between the placebo and dual-channel groups was somewhat surprising. It might have been due in part to the short treatment duration. In addition, the psychological placebo effects might also reduce the differences between the two groups, which suggested that the psychological rehabilitation was equally important with the function rehabilitation. Furthermore, the results of correlation analysis between changes on motor performance showed that the improvement of activities of daily living was closely related to the recovery of motor function and balance.

These findings are similar to those of some studies which used different ambulation training technologies. Lee and his colleagues [31] designed a 4-week randomized, controlled study to observe the effect of an electromechanical gait training on subjects with subacute stroke. The results showed that the electromechanical gait training combined with conventional FES improved FAC results significantly better than conventional gait training or electromechanical gait training alone. A group led by Alon [33] treated 10 chronic stroke subjects with motorized cycling. After 8 weeks of training, walking ability improved significantly. Both of those studies focused on task-specific gait training, upright or seated, while this study applied multichannel FES in the side-lying position, yet the gains in ambulation ability were similar.

How FES facilitates functional recovery in the affected limb remains unclear. Several studies have provided some evidence of brain plasticity in the lesional hemisphere which might be related to electrical stimulation [34–36]. Adkins has reported [34] that direct stimulation of the cortex promotes functional recovery and increases synaptic density in rats after simulated schemic stroke, which suggests that cortex stimulation might influence synaptic structural plasticity. A group led by Tarkka [35] found faster corticospinal conduction and new muscular responses in the primary motor cortex following the application of functional electrical therapy to the paretic upper limbs of subjects with chronic stroke. Here again the observed improvements might relate to brain plasticity induced by FES. The four-channel FES based on a normal gait pattern tested in this study, applied with the patient fully supported, allowed the subjects to perform walking movements earlier when they still were unable to stand or walk independently. Walking is a task-specific and repetitive functional activity, so early and repeated exercise may better promote motor relearning. Since the stimulation pattern of the four-channel FES is similar to the timing of a normal gait cycle, repeated stimulation may constitute meaningful sensory input and visual feedback motor information to the impaired brain, which may be important to functional recovery. It was not possible to perform either neurophysiological or neuroimaging studies to observe plastic changes in the brain during this intervention, but with the development of techniques such as diffusion tensor imaging, functional MRI and transcranial magnetic stimulation may be possible to obtain solid evidence for functional changes in the brain following FES.

This may be the first published study to compare the effects of multichannel FES with those of traditional FES stimulating ankle dorsiflexion alone. In the design of the study, here are some points that need to be explained. First, the study was applied in the side-lying position, which differed from the previous upright or walking function position, because the subjects enrolled in the study all had early stroke and most of them could not walk; the study thus used the side-lying position to reduce the weight of affected lower limb. Second, the subjects in a Brunnstrom stage of III were excluded owing to muscle spasms, and others in a Brunnstrom stage of V or VI also were not enrolled for better walking function, so generalizing the results to all of the recovery stages might not be appropriate. Third, the subjects in the placebo group usually questioned their treatment and even refused to continue because they had no feeling when they received treatment. At this situation, the physician should make some explanation to remove subjects' doubt and make them continue in the study.

In addition, there were also some limitations in the study. The sample was not large enough and the rate of dropouts was high at the follow-up time due to the limited research time and inconvenient activities, which might limit the effect of four-channel FES and decrease some subtle differences when compared with the other two groups. And although many scales were applied for the outcome measurement in the study, there still lacked some objective measurements (such as diffusion tensor imaging or motor evoked potentials) of motor performance improvement, and they will be the focus in our further study. Despite these limitations, the results provide a foundation for further exploring and developing alternative forms of intelligent FES for subjects with early stroke.

## 5. Conclusions

Four-channel FES and dual-channel FES can both improve motor function, balance, walking ability, and performance in the activities of daily living in subjects with early ischemic stroke. The training effects are sustained for at least 3 months after treatment. Compared with dual-channel FES stimulating ankle dorsiflexion only, four-channel FES based on a normal gait pattern may be a more effective approach. A larger sample and longer treatment period are necessary to definitively demonstrate its greater efficacy.

## Figures and Tables

**Table 1:** Subject characteristics.

	Four-channel	Placebo	Dual-channel
	(*n* = 16)	(*n* = 15)	(*n* = 14)
Age (years)	63.4 ± 10.6	67.0 ± 9.0	64.6 ± 8.3
Gender (male/female)	8/8	8/9	8/6
Brunnstrom (I/II/IV)	1/4/11	1/6/8	1/4/9
Time since onset (days)	41.3 ± 29.4	41.5 ± 20.4	41.6 ± 22.1

Four-channel, placebo, and dual-channel denote groups which received four-channel FES, four-channel FES placebo, and dual-channel FES, respectively. Value: mean ± SD.

**Table 2:** Comparison of outcomes measurements among the 3 groups.

Variable	Group	Week 0	Week 1	Week 2	Week 3	Followup
FMA	Four-channel	14.5 ± 5.1	18.7 ± 4.9^##^	20.6 ± 5.1^##^	21.8 ± 4.9^##^	23.3 ± 4.9
	Placebo	14.8 ± 5.6	16.6 ± 6.2^##^	17.9 ± 5.5^##^	18.2 ± 5.4^##^	19.5 ± 6.2
	Dual-channel	14.3 ± 5.1	15.9 ± 5.1^#^	16.8 ± 5.5^##^	17.3 ± 5.6^##∗^	19.3 ± 6.4

PASS	Four-channel	18.9 ± 8.8	26.6 ± 5.5^##^	28.9 ± 4.8^##^	29.8 ± 4.3^##^	30.9 ± 3.4
	Placebo	20.4 ± 9.1	22.1 ± 8.9^##^	23.8 ± 8.2^##^	24.1 ± 8.1^##∗^	25.7 ± 8.2
	Dual-channel	19.6 ± 10.1	22.0 ± 9.1^##^	24.5 ± 8.4^##^	25.4 ± 8.6^#^	26.8 ± 8.5

BBS	Four-channel	17.2 ± 14.9	30.4 ± 14.9^#^	37.6 ± 14.0^#^	39.1 ± 13.5^#^	42.7 ± 11.2
	Placebo	16.5 ± 16.7	21.1 ± 18.0^#^	24.4 ± 18.1^#∗^	25.4 ± 18.3^#∗^	29.3 ± 18.7∗
	Dual-channel	16.3 ± 16.3	19.5 ± 16.8^#^	24.8 ± 15.6^#∗^	27.4 ± 16.1^#^	31.9 ± 18.6

MBI	Four-channel	48.9 ± 23.8	65.7 ± 20.3^#^	76.3 ± 15.6^#^	80.3 ± 16.5^#^	87.4 ± 15.2
	Placebo	48.5 ± 21.7	58.0 ± 22.3^#^	64.3 ± 19.9^#^	66.7 ± 19.1^#∗^	72.9 ± 21.9∗
	Dual-channel	48.1 ± 22.6	55.3 ± 21.8^#^	62.5 ± 19.5^#∗^	64.6 ± 17.8^#∗^	75.8 ± 21.8

FAC	Four-channel	1.1 ± 1.4	1.5 ± 1.4	2.4 ± 1.5	2.6 ± 1.6	3.5 ± 1.5
	Placebo	0.8 ± 1.3	1.0 ± 1.4	1.5 ± 1.4	1.5 ± 1.3	2.3 ± 1.9
	Dual-channel	0.7 ± 1.1	0.8 ± 1.1	1.1 ± 1.1	1.4 ± 1.1	2.4 ± 1.8

Value: mean ± SD. ^#^ *P* < 0.05, ^##^ *P* < 0.01 indicate significant differences when comparing the score of all outcomes for week 1 to followup at 3 months after the 3-week treatment ended with week 0 within-group. *P < 0.05 indicates significant difference when compared with the four-channel group.

## References

[1] Wade DT, Wood VA, Heller A, Maggs J, Langton Hewer R (1987). Walking after stroke: measurement and recovery over the first 3 months. *Scandinavian Journal of Rehabilitation Medicine*.

[2] Friedman PJ (1990). Gait recovery after hemiplegic stroke. *International Disability Studies*.

[3] Chen G, Patten C, Kothari DH, Zajac FE (2005). Gait differences between individuals with post-stroke hemiparesis and non-disabled controls at matched speeds. *Gait & Posture*.

[4] Sullivan KJ, Knowlton BJ, Dobkin BH (2002). Step training with body weight support: effect of treadmill speed and practice paradigms on poststroke locomotor recovery. *Archives of Physical Medicine and Rehabilitation*.

[5] Stein RB, Chong S, Everaert DG (2006). A multicenter trial of a footdrop stimulator controlled by a tilt sensor. *Neurorehabilitation and Neural Repair*.

[6] Liberson WT, Holmquest HJ, Scot D, Dow M (1961). Functional electrotherapy: stimulation of the peroneal nerve synchronized with the swing phase of the gait of hemiplegic patients. *Archives of physical medicine and rehabilitation*.

[7] Sabut SK, Sikdar C, Mondal R, Kumar R, Mahadevappa M (2010). Restoration of gait and motor recovery by functional electrical stimulation therapy in persons with stroke. *Disability and Rehabilitation*.

[8] Sabut SK, Sikdar C, Kumar R, Mahadevappa M (2011). Functional electrical stimulation of dorsiflexor muscle: effects on dorsiflexor strength, plantarflexor spasticity, and motor recovery in stroke patients. *NeuroRehabilitation*.

[9] Hara Y, Ogawa S, Tsujiuchi K, Muraoka Y (2008). A home-based rehabilitation program for the hemiplegic upper extremity by power-assisted functional electrical stimulation. *Disability and Rehabilitation*.

[10] Kesar TM, Perumal R, Jancosko A (2010). Novel patterns of functional electrical stimulation have an immediate effect on dorsiflexor muscle function during gait for people poststroke. *Physical Therapy*.

[11] Flansbjer U, Downham D, Lexell J (2006). Knee muscle strength, gait performance, and perceived participation after stroke. *Archives of Physical Medicine and Rehabilitation*.

[12] Bowden MG, Balasubramanian CK, Neptune RR, Kautz SA (2006). Anterior-posterior ground reaction forces as a measure of paretic leg contribution in hemiparetic walking. *Stroke*.

[13] Balasubramanian CK, Bowden MG, Neptune RR, Kautz SA (2007). Relationship between step length asymmetry and walking performance in subjects with chronic hemiparesis. *Archives of Physical Medicine and Rehabilitation*.

[14] Stanic U, Acimovic-Janezic R, Gros N, Trnkoczy A, Bajd T, Kljajić M (1978). Multichannel electrical stimulation for correction of hemiplegic gait: methodology and preliminary results. *Scandinavian Journal of Rehabilitation Medicine*.

[15] Bogataj U, Gros N, Malezic M, Kelih B, Kljajic M, Acimovic R (1989). Restoration of gait during two to three weeks of therapy with multichannel electrical stimulation. *Physical Therapy*.

[16] Daly JJ, Roenigk K, Holcomb J (2006). A randomized controlled trial of functional neuromuscular stimulation in chronic stroke subjects. *Stroke*.

[17] Jensen CV (1991). A computer program for randomizing patients with near-even distribution of important parameters. *Computers and Biomedical Research*.

[18] Sze K, Wong E, Or KH, Lum CM, Woo J (2000). Factors predicting stroke disability at discharge: a study of 793 Chinese. *Archives of Physical Medicine and Rehabilitation*.

[19] Yan T, Hui-Chan CWY, Li LSW (2005). Functional electrical stimulation improves motor recovery of the lower extremity and walking ability of subjects with first acute stroke: a randomized placebo-controlled trial. *Stroke*.

[20] Meyer ARF, Jaasko L, Leyman I, Olsson S, Steglind S (1975). The post stroke hemiplegic patient. I. A method for evaluation of physical performance. *Scandinavian Journal of Rehabilitation Medicine*.

[21] Benaim C, Pérennou DA, Villy J, Rousseaux M, Pelissier JY (1999). Validation of a standardized assessment of postural control in stroke patients: the Postural Assessment Scale for Stroke patients (PASS). *Stroke*.

[22] Mao HF, Hsueh IP, Tang PF, Sheu CF, Hsieh CL (2002). Analysis and comparison of the psychometric properties of three balance measures for stroke patients. *Stroke*.

[23] Bateman A, Culpan FJ, Pickering AD, Powell JH, Scott OM, Greenwood RJ (2001). The effect of aerobic training on rehabilitation outcomes after recent severe brain injury: a randomized controlled evaluation. *Archives of Physical Medicine and Rehabilitation*.

[24] Blum L, Korner-Bitensky N (2008). Usefulness of the Berg Balance Scale in stroke rehabilitation: a systematic review. *Physical Therapy*.

[25] Demeurisse G, Demol O, Robaye E (1980). Motor evaluation in vascular hemiplegia. *European Neurology*.

[26] Collen FM, Wade DT, Bradshaw CM (1990). Mobility after stroke: reliability of measures of impairment and disability. *International Disability Studies*.

[27] Eakin P, Baird H (1995). The community dependency index: a standardized assessment of need and measure of outcome for community occupational therapy. *The British Journal of Occupational*.

[28] Hachisuka K, Ogata H, Ohkuma H, Tanaka S, Dozono K (1997). Test-retest and inter-method reliability of the self-rating Barthel index. *Clinical Rehabilitation*.

[29] Kesar TM, Perumal R, Reisman DS (2009). Functional electrical stimulation of ankle plantarflexor and dorsiflexor muscles: effects on poststroke gait. *Stroke*.

[30] Embrey DG, Holtz SL, Alon G, Brandsma BA, McCoy SW (2010). Functional electrical stimulation to dorsiflexors and plantar flexors during gait to improve walking in adults with chronic hemiplegia. *Archives of Physical Medicine and Rehabilitation*.

[31] Lee HJ, Cho KH, Lee WH (2013). The effects of body weight support treadmill training with power-assisted functional electrical stimulation on functional movement and gait in stroke patients. *American Journal of Physical Medicine and Rehabilitation*.

[32] McCabe JP, Dohring ME, Marsolais EB (2008). Feasibility of combining gait robot and multichannel functional electrical stimulation with intramuscular electrodes. *Journal of Rehabilitation Research and Development*.

[33] Alon G, Conroy VM, Donner TW (2011). Intensive training of subjects with chronic hemiparesis on a motorized cycle combined with functional electrical stimulation ( fes ): a feasibility and safety study. *Physiotherapy Research International*.

[34] Adkins DL, Hsu JE, Jones TA (2008). Motor cortical stimulation promotes synaptic plasticity and behavioral improvements following sensorimotor cortex lesions. *Experimental Neurology*.

[35] Tarkka IM, Pitkänen K, Popovic DB, Vanninen R, Könönen M (2011). Functional electrical therapy for hemiparesis alleviates disability and enhances neuroplasticity. *The Tohoku Journal of Experimental Medicine*.

[36] Barsi GI, Popovic DB, Tarkka IM, Sinkjær T, Grey MJ (2008). Cortical excitability changes following grasping exercise augmented with electrical stimulation. *Experimental Brain Research*.

